# Practical Notes

**Published:** 1890-09

**Authors:** 


### PRACTICAL NOTES.

FOR INFANTILE PNEUMONIA.

℞.—Quininæ bisulph . . . . ℈j.
Ol. theobromæ . . . . . . ℥j.

M.—et in supposit. No. iv, div.

S. One every eight hours. Also, paint the back of chest with iodine and envelop in flaxseed jacket. Internally, give digitalis or ergot, in small doses. —*Waugh.*

FOR CHOLERA MORBUS.

℞.—Tinct. capsici. . . . . . . ʒij.

Ol. cajuputi. . . . . . . ʒij.

Chloroformi. . . . . . . ʒj.

Æther. fort. . . q. s. ad. fʒij.

M.—S. ʒj to be taken, without water, every half hour till relieved. —*Waugh.*

FOR SUMMER DIARRHŒA IN CHILDREN.

℞.—Zinci sulphocarbolat. . . gr. ss.
Bismuth subnit. . . . . . gr. ij.
Codeinæ . . . . . . . . . gr. 1-24.

M.—Fiat tablet, No. j.

S. To be taken every one to three hours.

For children after the second year. —*Waugh.*

FOR SUMMER DIARRHŒA IN ADULTS.

℞.—Tinct. hydrastis . . . . . . . ℥j.

Syr. rhei. aromat. . . . . . . ʒv.

Potass. carb. *vel* nitrat. . . ʒss.

M.—S. ℥ss every four hours. —*Waugh.*

FOR AMENORRHŒA.

℞.—Hydrargyri bichlorid. . . gr. iv.
Sodii arseniatis. . . . . gr. ijss.
Strychninæ sulphat. . . . gr. ss.
Potassii carb. pur.,
Ferri sulphat. exsic. . aa gr. lx.

M.—et div. in pil. No. lx.

S. One pill after each meal.—Winton, *Occid. Med. Times.*

—*Times and Register.*

TO REMOVE SUMMER FRECKLES.

℞. White precipitate,
Subnitrate of bismuth . . aa ʒ i.
Glycerite of starch . . . ℥ iv. M.

Every second day apply a coating of this preparation to the freckles.

Washing the affected parts with the following lotion mornings and evenings will also suffice to remove them:

℞ Sulpho-carbolate of zinc . . ʒ i.

Glycerin . . . . . . . . . . . f ℥ ii.

Alcohol . . . . . . . . . . . f ʒ i.

Orange-flower water . . . f ℥ iss.

Rose water . . . q. s. ad ℥ f viii.

M. *'L' Union Medicale.*

ECZEMA OF THE HEAD.

Wash the head with soap and water, and apply the following ointment:

℞. Acid. salicyl. . . . . . grs. xxv.

Tinct. benzoin. . . . . . . . ʒ i.

Vaselini . . . . . . . . . . . ℥ i.

M.

FOR OZŒNA.

The nasal injections of warm sulphur water and the following solution:

℞. Creoline . . . . . . . 1 gramme.
Pure alcohol . . . 120 grammes.

M. Sig. Put a teaspoonful of this into a quart of warm water, and use the nasal douche, one-half at a time only. In regard to nasal douches, it is now thought best not to inject more than a pint at a sitting, and to instruct the patient to keep the head erect, and in blowing the nose only to blow out one side at a time, keeping the other closed; this prevents strain on the vessels, and resulting hemorrhage.

FOR TAN AND FRECKLES.

Dr. Chevasses' preparation for tan, freckles, pimples, etc.:

℞. Rose water . . . . . . 6 ounces.
Glycerine . . . . . . . 1-2 ounce.
Bitter almond water,
Tinct. benzoin . . aa 2 1-2 drams.
Borax . . . . . . . 1 1-2 drams.

Rub the borax with the glycerine, gradually adding the rose and almond water; lastly add the tincture benzoin, agitating constantly. Apply night and morning.—*Dietetic Gazette.*

FOTHERGILL'S ANTI-RHEUMATIC PILLS.—The late Dr. Fothergill used the following combination in a large proportion of his cases of chronic rheumatism:

| | | |
|---|---|---|
| ℞. | Arsenious acid . . . . . . | 3 grains. |
| | Powdered guaic . . . . | 3 drachms. |
| | Powdered capsicum . . . | 30 grains. |
| | Pill of aloes and myrrh . . | 3 drachms. |

Mix, and divide in 120 pills. One pill was ordered three times a day, in connection with a diet rich in fatty foods. Also, a general tonic treatment was in most cases found advisable at the outset.

TREATMENT OF TYMPANITES.—

| | | |
|---|---|---|
| ℞. | Naphthol<br>Carbonate of Magnesia<br>Charcoal | of each 1 1-2 drachms. |
| | Essence of peppermint . . . | 10 drops. |

Make into twelve powders, and take one every two hours until relief is obtained.—*Revue Obstetricale et Gynecologique*, July, 1890.

CREOLIN FOR PRURITUS VULVŒ.—

| | |
|---|---|
| Creolin . . . . . . . | 3 to 5 parts. |
| Linseed oil . . . . . . | 100 parts. |

Apply with friction to the affected parts every four or five hours.—*Revue Obstetricale et Gynecologique*, July, 1890.

FORMULA FOR THE ADMINISTRATION OF CREOSOTE IN PHTHISIS.—

| | | |
|---|---|---|
| ℞. | Creosote . . . . . . . . | 30 grains. |
| | Rum . . . . . . . . . | 1 1-2 ounces. |
| | Syrup of tolu . . . . . . | 1 ounce. |
| | Distilled water . . . . | 2 ounces. |

A dessertspoonful twice or thrice a day in a wine glassful of water, to persons having a tubercular tendency.—*L' Union Medicale*, July 5, 1890.

